# Gaps in the prevention of perinatal transmission of hepatitis B virus between recommendations and routine practices in a highly endemic region: a provincial population-based study in China

**DOI:** 10.1186/1471-2334-12-221

**Published:** 2012-09-17

**Authors:** Yali Hu, Shu Zhang, Chao Luo, Qilan Liu, Yi-Hua Zhou

**Affiliations:** 1 Department of Obstetrics and Gynecology, Nanjing Drum Tower Hospital, Nanjing University Medical School, Nanjing, China; 2 Jiangsu Family Planning Institute, Nanjing, China; 3 Departments of Laboratory Medicine and Infectious Diseases, Nanjing Drum Tower Hospital, Nanjing University Medical School, Nanjing, China; 4 Jiangsu Key Laboratory for Molecular Medicine, Nanjing University Medical School, Nanjing, China

**Keywords:** Hepatitis B virus, Perinatal infection, Immunoprophylaxis, Gaps

## Abstract

**Background:**

Hepatitis B virus (HBV) infection is endemic in China; perinatal transmission is the main source of chronic HBV infection. Simultaneous administration of hepatitis B immune globulin (HBIG) and hepatitis B vaccine is highly effective to prevent perinatal transmission of HBV; however, the effectiveness also depends on full adherence to the recommended protocols in daily practice. In the present investigation, we aimed to identify gaps in immunoprophylaxis of perinatal transmission of HBV between recommendations and routine practices in Jiangsu Province, China.

**Methods:**

Totally 626 children from 6 cities and 8 rural areas across Jiangsu Province, China, born from February 2003 to December 2004, were enrolled; 298 were born to mothers with positive hepatitis B surface antigen (HBsAg) and 328 were born to HBsAg-negative mothers. Immunoprophylactic measures against hepatitis B were retrospectively reviewed for about half of the children by checking medical records or vaccination cards and the vaccine status was validated for most of children.

**Results:**

Of 298 children born to HBV carrier mothers, 11 (3.7%) were HBsAg positive, while none of 328 children born to non-carrier mothers was HBsAg positive (P < 0.01). The rates of anti-HBs ≥ 10 mIU/ml in children of carrier and non-carrier mothers were 69.5% and 69.2% respectively (P = 0.95). The hepatitis B vaccine coverage in two groups was 100% and 99.4% respectively (P = 0.50), but 15.1% of HBV-exposed infants did not receive the timely birth dose. Prenatal HBsAg screening was performed only in 156 (52.3%) of the carrier mothers. Consequently, only 112 (37.6%) of HBV-exposed infants received HBIG after birth. Furthermore, of the 11 HBV-infected children, only one received both HBIG and hepatitis B vaccine timely, seven missed HBIG, two received delayed vaccination, and one missed HBIG and received delayed vaccination.

**Conclusions:**

There are substantial gaps in the prevention of perinatal HBV infection between the recommendations and routine practices in China, which highlights the importance of full adherence to the recommendations to eliminate perinatal HBV infection in the endemic regions.

## Background

Chronic hepatitis B virus (HBV) infection is a major public health problem worldwide, particularly in Asia and Africa, and Asian communities in low or high HBV endemic areas [1,2]. Prevention of perinatal HBV infection from HBV carrier mothers to their infants is critical to eliminate chronic hepatitis B since as high as 90% of the neonatal infections will become chronically infected. Simultaneous administration with one dose (0.5–1 ml, 100–200 IU) of hepatitis B immune globulin (HBIG) within 12 hours after birth and three doses of hepatitis B vaccine on a 0-, 1-, and 6-month schedule, which has been adopted by many countries as well as China as the recommended immunoprophylaxis for infants born to HBV carrier mothers, is highly effective to prevent perinatal HBV infections [3-5]. Up to 2008, 177 countries included the hepatitis B vaccine into their national infant immunization programs [6]. The Chinese government formally integrated hepatitis B vaccine into its Expanded Program on Immunization (EPI) in 2002, and since then all newborns in China may receive three doses of hepatitis B vaccine without charge [7,8].

The protective effect of a vaccine is not only dependent on its quality, but also on full adherence to the recommended protocols in daily practice. In developed countries, the vaccination schedule against hepatitis B has been followed well in the routine practices and the protective efficiency was 90–100% in infants born to HBV carrier mothers, as high as the protective rates achieved in the clinical trials [9-12]. In developing countries like China, where HBV infection is highly endemic, however, the protective efficiency in daily practice has been less studied. In the present study, we surveyed the administration of hepatitis B vaccine and HBIG and measured the serologic markers in infants born to HBV carrier mothers based on the pregnant women population in Jiangsu province, China, to determine whether there are gaps in the immunoprophylaxis against perinatal HBV transmission between national recommendations and the routine practices after the integration of hepatitis B vaccine into the EPI of China.

## Methods

### Study design and subjects

**Figure 1:**
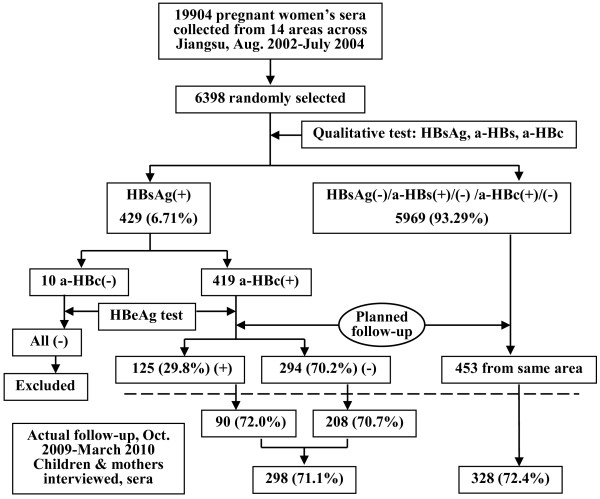
**Flow diagram of study subjects.** HBsAg, hepatitis B surface antigen; a-HBs, antibody against HBsAg; a-HBc, antibody against hepatitis B core antigen. HBeAg, hepatitis B e antigen.

During August 2002 to July 2004, serum samples from 19 904 pregnant women aged 20–42 years, at 15–20 weeks of gestation, from 6 cities (urban) and 8 counties (rural area) across Jiangsu Province, China, were collected in a study on the provincial prevalence of birth defects [13]. The samples were stored at −30°C. These pregnant women had been selected to represent the pregnant women population in Jiangsu and all delivered their infants in hospitals. Jiangsu Province is located in the east of China, and is one of the six financially prosperous provinces in China and has the densest population with more than 76 million inhabitants. There are approximately 500 000 live births per year. Recently, we retrospectively measured the HBV serologic markers in the 6398 sera, which were randomly selected from above samples, and 429 (6.7%) were positive for hepatitis B surface antigen (HBsAg) [14]. Of the 429 HBsAg positive sera, 10 were negative for antibody against hepatitis B core antigen (anti-HBc) and either hepatitis B e antigen (HBeAg) or anti-HBe. The positivity of HBsAg in these 10 samples was validated by retesting with Architect HBsAg Reagents (Abbott, North Chicago, USA) and by detection of HBV DNA [14]; we speculated that these 10 women were in the incubation period of HBV infection when the sera were collected. Thus, we excluded these 10 women from the study and planned to follow up the children of 419 HBsAg positive women, of whom 125 (29.8%) were also positive for HBeAg [14]. Because of the “one family one child policy” in China, the target population was the 419 children born from February 2003 to December 2004 (Figure 1). As control subjects, 453 children born to HBsAg negative mothers in the same areas and same period of time were randomly selected (Figure 1).

During October 2009 and March 2010, we invited these mothers and their children to participate in the present investigation as an “add-on” part of the project on the provincial prevalence of birth defects [13]. Each attending mother was asked to complete a questionnaire, which included demographic data of the mother and her child, screening for HBsAg during pregnancy, and use of hepatitis B vaccine and HBIG in the child. Nearly half of the HBsAg screening in pregnant women and the HBIG administration in infants were validated by checking the hospital discharge records. The use of hepatitis B vaccine was evidenced in 83.7% children by the vaccination record cards. Furthermore, ~3 ml of blood was collected from each child after obtaining the consent from the child’s mother.

This study followed the ethical guidelines of the Declaration of Helsinki, and was approved by the institutional review boards of Nanjing Drum Tower Hospital and Jiangsu Family Planning Institute. The pregnant women consented to participate in the birth defect study conducted from August 2002 to July 2004 [13] and their serum samples were used in this study; the mothers consented to be interviewed and consented to their children's participation in this follow-up study.

### Laboratory procedures

All serum samples were tested by commercial ELISA kits for the presence of HBsAg, antibody to HBsAg (anti-HBs), and anti-HBc. HBsAg was tested with an ELISA kit (Huakang Biotech, Shenzhen, China), and anti-HBs and anti-HBc were quantitatively tested with microparticle enzyme immunoassay (AxSYM AUSAB, Abbott, North Chicago, USA). Quantitative levels of anti-HBs were expressed in international units.

### Definitions and statistical analysis

HBV infection was defined by the presence of HBsAg. The presence of anti-HBc in the absence of HBsAg represented resolved HBV infection. Timely vaccination referred to receipt of the first dose of the vaccine within 24 hours after birth.

Data were analyzed using SPSS version 13 (SPSS Inc., Chicago, USA). Statistical comparisons of continuous variables between maternal HBsAg positive and negative groups were analyzed by *t*-test. A *χ*^2^ test was used to analyze and compare categorical data. A *P* value of <0.05 was considered statistically significant.

## Results

### Effect of routine hepatitis B immunization on the prevalence of HBV in infants of HBsAg positive mothers

Of the 419 invited mothers with positive HBsAg, 298 (71.1%) mothers and their children participated in the study, while 328 (72.4%) of the 453 invited mothers who were negative for HBsAg and their children attended the investigation (Figure 1); the follow-up rates were comparable in these two groups (P > 0.05). Additionally, the follow-up rate of children born to HBV carrier mothers with positive HBeAg was also similar to that of children of carrier mothers with negative HBeAg (72.0% vs. 70.7%, Figure 1). The demographic data, vaccination coverage, status of HBV infection, and anti-HBs responses in children are shown in Table 1. Almost all infants in the both groups were vaccinated and the positive rates of anti-HBs were comparable. Of the children born to HBsAg positive mothers, 11 (3.7%) were HBsAg positive, demonstrating the HBV infection, and 16 (5.4%) were HBsAg negative but anti-HBc positive, indicating past resolved infection, whereas none of the children born to HBsAg negative mothers was infected with HBV and only 0.9% had the resolved infection.

**Table 1:** Demography, status of HBV infection and anti-HBs in children of mothers who were positive or negative for HBsAg

	**Maternal HBsAg at gestation**		***P*value**
	**Positive (n = 298)**	**Negative (n = 328)**	
Age (year)	6.22 ± 0.82	6.25 ± 0.76	0.70
Male (%)	52.4	57.9	0.16
HBIG use (%)	112 (37.6)	0	–
Vaccination (%)	298 (100)	326 (99.4)	0.50
HBsAg + (%)	11 (3.7)	0	< 0.01
HBsAg–/anti-HBc + (%)	16 (5.5)	3 (0.9)	0.01
Anti-HBs > 10 mIU/ml (%)	207 (69.5)	227 (69.2)	0.95

### Administration of immunoprophylaxis in infants of HBsAg positive mothers

Since recommended immunoprophylaxis against hepatitis B in infants born to HBsAg positive mothers requires the timely use of hepatitis B vaccine and HBIG, we particularly paid attention to collecting the data about the preventive measures used in the routine practices (Table 2). Although prenatal HBsAg screening is recommended for all pregnant women in China, the test was not done in nearly a third of the pregnant women in cities and more than half in rural areas. Consequently, only 37.6% of the HBV-exposed infants received HBIG after birth, and the use of HBIG in HBV-exposed infants was less frequently in rural areas than in cities (55.7% vs. 32.0%, P <0.001). In other words, 62.4% of the infants born to HBV carrier mothers were not administered with HBIG in Jiangsu Province from February 2003 to December 2004. This was a substantial gap in the prevention of HBV infection. Additionally, the first dose of the vaccine was delayed in 15.1% of the HBV-exposed infants.

**Table 2:** Immunoprophylaxis in infants of HBsAg positive mothers in Jiangsu, 2002–2004

	**Total n = 298 (%)**	**Urban n = 70 (%)**	**Rural n = 228 (%)**	***P*value**
Prenatal HBsAg screening	156 (52.3)	49 (70.0)	107 (46.9)	< 0.001
Use of HBIG for infants	112 (37.6)	39 (55.7)	73 (32.0)	< 0.001
Use of HBV vaccine for infants	298 (100)	70 (100)	228 (100)	
Timely birth dose	253 (84.9)	60 (85.7)	193 (84.6)	0.828
Fewer than three doses	21 (7.0)	3 (4.3)	18 (7.9)	
Three doses but not on schedule	42 (14.1)	12 (17.1)	30 (13.2)	
Three doses on time	235 (78.9)	54 (77.1)	181 (79.4)	

### Inadequate implementation of the immunoprophylaxis responsible for most HBV infection in children

Of the 298 children born to HBV carrier mothers, 11 were infected with HBV and 16 experienced resolved infections (Table 1). To clarify the factors responsible for the infections, we analyzed the HBeAg status in their mothers and the immunoprophylactic measures used in these children. All 11 children infected with HBV and 12 of 16 children with the resolved infection were born to HBeAg positive mothers. These results are in agreement with the previous reports that children born to HBV carrier mothers with positive HBeAg are much more frequently to be infected than those born to HBeAg negative HBV carrier mothers [15,16]. On the other hand, of the 11 children infected with HBV, only one received timely administration of both HBIG and hepatitis B vaccine, and 10 others did not receive HBIG or received delayed hepatitis B vaccine (Table 3). Of the 16 children with the resolved infections, 9 were not administered with HBIG and one was given the first dose of vaccine 40 days after birth (Table 3). The results demonstrated that the infections in children born to HBV carrier mothers were mostly attributed to the inadequate implementation of the immunoprophylaxis.

**Table 3:** Immunoprophylaxis used in children with HBV infection or resolved infection*

Child**	**HBIG (doses)**	**Vaccination**			
		**Doses**	**1st**	**2nd (m)**	**3rd (m)**
Children with positive HBsAg					
A	2	3	24 h	1	6
B	2	3	2 m	5	unknown
C	2	3	1 m	2	7
D	0	3	7 d	1	6
E	0	3	24 h	1	6
F	0	3	24 h	1	6
G	0	3	24 h	1	6
H	0	3	24 h	1	6
I	0	3	24 h	1	6
J	0	3	24 h	1	6
K	0	3	24 h	1	6
Children with negative HBsAg and positive anti-HBc					
a	2	3	24 h	1	6
b	2	3	24 h	1	6
c	2	3	24 h	1	6
d	1	3	24 h	1	6
e	1	3	24 h	1	6
f	1	3	24 h	1	6
g	1	3	24 h	1	6
h	0	3	24 h	1	6
i	0	3	24 h	1	6
j	0	3	24 h	1	6
k	0	3	24 h	1	6
l	0	3	40 d	2	5
m	0	2	24 h	1	–
n	0	3	24 h	1	6
o	0	3	24 h	1	6
p	0	3	24 h	1	6

*Mothers of children A–K and a–l were positive for both HBsAg and HBeAg, and mothers of children m–p were positive for HBsAg and negative for HBeAg.

**Children a–j and l–n were also positive for anti-HBs, and children k, o, and p were negative for anti-HBs.

## Discussion

In the present population-based study, we found that, although most children of HBV carrier mothers were protected against chronic HBV infection after the introduction of the universal immunization, there were considerable gaps in the immunoprophylaxis of perinatal HBV infection between the routine practices and national recommendations in China. Such gaps may also exist in other developing countries since only 23.7% of the 8–10-year-old children in East Java, Indonesia, were anti-HBs positive after introduction of universal vaccination program [17] and the vaccine coverage in remaining quilombo communities in Central Brazil is suboptimal [18]. Therefore, the significance of full compliance with the recommended procedures to prevent perinatal HBV transmission should be emphasized in China as well as other developing countries.

Prenatal HBsAg screening has been recommended in all pregnant women in China since the late of 1980s, however, the actual screening rate in the present investigation was only 52.3%, far from the rates of 90–99% in developed countries [19-22]. The gap is particularly substantial in the rural areas as only 46.9% of pregnant women underwent the prenatal screening. Since all investigated women had experienced prenatal examinations and delivered their children in hospitals, we considered that the low HBsAg screening rate was largely attributed to the health care providers’ unawareness of the importance of the screening. We can imagine that the adherence to preventive measures may be worse in infants of mothers delivering at home in remote rural areas of China. Thus, more intensive information about the importance of preventing mother-to-infant transmission of HBV should be provided to health care providers as well as pregnant women to increase prenatal HBsAg screening.

The administration of prophylactic measures against hepatitis B in HBV-exposed infants was far from optimal in China. Although all the infants received hepatitis B vaccine, the birth dose was delayed in 15.1% of the newborns and three doses were not completed in 7% of them. Furthermore, HBIG was used only in 37.6% of the high risk infants. The untimely use of the birth dose vaccine was apparently due to the oversight of the health care providers since it is recommended that the birth dose vaccine be given within 24 hours after birth. The low usage of HBIG might be caused by several reasons, first, unavailability of HBIG in some hospitals because these hospitals do not have specific policies on preventing perinatal HBV infection; second, the lack of knowledge on the standard prophylaxis in the health care providers; third, neglecting the use of HBIG by some health care providers since the implementation of universal vaccination against hepatitis B, whatever the status of the mother, might have been confusing for those not specialized in HBV infection; and fourth, also most important, unknown HBsAg status in the pregnant women before birth because of the low rate of prenatal HBsAg screening. Unlike the recommendation in USA, in which infants born to women with unknown HBsAg status are administered with both HBIG and hepatitis B vaccine [23], the policy in China is that only infants born to known HBV carrier mothers receive passive-active immunoprophylaxis.

Although only 3.7% of HBV-exposed infants in this investigation were infected with HBV, our findings indicate that there is still a room to increase the protection conferred by the vaccination against hepatitis B, because the failure of the protection is largely due to the inappropriate administration of the recommended prophylactic procedures in the routine practices (Table 3). Additionally, recent studies show that in developed countries, 98–100% of the HBV-exposed infants were protected against the chronic infection after passive-active immunoprophylaxis had been strictly followed [9-12]. This highlights the importance of the full adherence to the standard immunoprophylaxis in the prevention of perinatal HBV infections.

There are some limitations in the present study. First, the overall follow-up rate (71.1%) in HBV-exposed children was not high. However, it is less likely that the HBsAg positive rate in the children was biased due to this reason, since a comparable follow-up rate (72%) in children born to HBeAg positive carrier mothers, whose infants are more prone to be infected, was achieved. Second, the perinatal HBV infection in this study was examined at the age of 5–7 years, rather than at 12 months old. However, spontaneous HBsAg loss in children perinatally infected is very low [24], and novel HBV infection in vaccinated children rarely occurs [25]. Thus, the positive rate of HBsAg in children at ages of 5–7 years whose mothers are infected with HBV may essentially represent the perinatal infection. Third, the measures in the routine practices investigated in this survey were implemented during 2002–2004 and not all the data on screening for HBsAg in pregnant women and administration of HBIG in infants were validated by medical records; the current status of services for infants of HBV carrier mothers could be improved. However, a recent preliminary survey in the prevention of perinatal HBV transmission among obstetric and gynecological medical staffs in China showed that there is insufficient training in the application of immunoprophylaxis, particularly in rural areas (unpublished data), suggesting there is a big room to improve the immunoprophylaxis against hepatitis B. On the other hand, there are two strengths in this study. One is that the study subjects were from 14 areas across Jiangsu; the data should be superior to those from a single center study. The other is that we conducted this study as the third party in a more objective manner.

Although the results in this study were derived from a province in China, we consider that the identified gaps also exist in most areas of China as Jiangsu is one of the six financially prosperous regions among the 31 provinces in Mainland China. We also consider that the data of the present study may basically reflect the scenario in some developing countries like China. Thus, a national organization dedicated to educating health care providers in delivery units and delivery hospitals about the knowledge of preventing perinatal HBV infection will be critical for filling the gaps.

## Conclusions

Despite the availability of effective immunoprophylaxis in the prevention of perinatal transmission of HBV for more than 20 years, there are substantial gaps between the recommendations and routine practices in China, which highlights the importance of full adherence to the recommendations to eliminate perinatal HBV infections in the endemic regions.

## Abbreviations

HBV, Hepatitis B virus; HBsAg, Hepatitis B surface antigen; HBIG, Hepatitis B immune globulin; HBeAg, Hepatitis B e antigen; Anti-HBs, Antibody against HBsAg; Anti-HBc, Antibody against hepatitis B core antigen; EPI, Expanded program on immunization.

## Competing interests

The authors declare that they have no conflict of interest.

## Authors’ contributions

YH and SZ participated in the design of the study, acquisition and interpretation of the data; CL and QL performed the laboratory procedures. SZ performed the statistical analysis. YHZ conceived of and designed the study, directed its implementation, interpreted data, and drafted the manuscript. All authors read and approved the final manuscript.

## Pre-publication history

The pre-publication history for this paper can be accessed here:

http://www.biomedcentral.com/1471-2334/12/221/prepub
